# The prognostic significance of the alterations of pulmonary hemodynamics in patients with pulmonary arterial hypertension: a meta-regression analysis of randomized controlled trials

**DOI:** 10.1186/s13643-021-01816-0

**Published:** 2021-10-30

**Authors:** Shih-Hsien Sung, Wan-Yu Yeh, Chern-En Chiang, Chi-Jung Huang, Wei-Ming Huang, Chen-Huan Chen, Hao-Min Cheng

**Affiliations:** 1 Division of Cardiology, Department of Internal Medicine, Taipei Veterans General Hospital, Taipei, Taiwan; 2 Department of Medicine, National Yang Ming Chiao Tung University, School of Medicine, Taipei, Taiwan; 3 Center for Evidence-based Medicine, Taipei Veterans General Hospital, No. 201, Sec. 2, Shih-Pai Road, Beitou District, Taipei, Taiwan; 4 General Clinical Research Center, Taipei Veterans General Hospital, Taipei, Taiwan; 5 Institute of Public Health and Community Medicine Research Center, National Yang Ming Chiao Tung University, School of Medicine, Taipei, Taiwan; 6 Department of Medical Education, Taipei Veterans General Hospital, Taipei, Taiwan

**Keywords:** Pulmonary arterial hypertension, Hemodynamics, Meta-regression, Meta-analysis

## Abstract

**Background:**

Hemodynamic assessment in patients with pulmonary arterial hypertension (PAH) is essential for risk stratification and pharmacological management. However, the prognostic value of the hemodynamic changes after treatment is less well established.

**Objectives:**

We investigated the prognostic impacts of the changes in hemodynamic indices, including mean pulmonary artery pressure (mPAP), pulmonary vascular resistance (PVR), right atrial pressure (RAP), and cardiac output index (CI). We conducted this systematic review with meta-regression analysis on existing clinical trials.

**Methods:**

We searched and identified all relevant randomized controlled trials from multiple databases. An analogous *R*^2^ index was used to quantify the proportion of variance explained by each predictor in the association with PAH patients’ prognosis. A total of 21 trials and 3306 individuals were enrolled.

**Results:**

The changes in mPAP, PVR, RAP, and CI were all significantly associated with the change in 6MWD (∆6MWD). The change in mPAP was with the highest explanatory power for ∆6MWD (*R*^2^ analog = 0.740). Additionally, the changes in mPAP, PVR, and CI were independently predictive of adverse clinical events. The change in mPAP had the highest explanatory power for the clinical events (*R*^2^ analog = 0.911). Furthermore, the change in PVR was with the highest explanatory power for total mortality of PAH patients (*R*^2^ analog = 0.612).

**Conclusion:**

Hemodynamic changes after treatment, including mPAP, PVR, CI, and RAP, were significantly associated with adverse clinical events or mortality in treated PAH patients. It is recommended that further studies be conducted to evaluate the changes in hemodynamic indices to guide drug titration.

**Systematic review registration:**

PROSPERO CRD42019125157

**Supplementary Information:**

The online version contains supplementary material available at 10.1186/s13643-021-01816-0.

## Introduction

Although there have been significant advances in pharmacological therapies in the past decade, pulmonary arterial hypertension (PAH) remains a progressive and fatal disease. The 2015 ESC/ERS Pulmonary Hypertension guidelines have strongly recommended comprehensive screening protocols for high-risk populations and subsequent early intervention [1]. In addition, upfront combination therapy and aggressive medical escalations were also suggested in the treatment of PAH patients. Given the variable long-term survival rates between patients, risk stratification has been endorsed in the clinical management of PAH. While the European guideline has proposed a risk prediction algorithm, comprising 9 measures [1], Benza et al. also computed a risk score calculator for 1-year survival in 504 individuals from the Registry to Evaluate Early and Long-term PAH Disease Management (REVEAL Registry) [2]. However, the routine clinical application was limited due to the complexity of these predictive algorithms. Hoeper et al., therefore, validated a simplified risk stratification strategy for mortality, including World Health Organization functional class (WHO Fc), 6-min walking distance (6WMD), brain natriuretic peptide or its N-terminal fragment, right atrial pressure (RAP), and cardiac index (CI) in a cohort of 1588 PAH patients [3]. Despite the existing prediction models, the prognostic significance of the changes of these parameters during treatment for patients with PAH has not been systematically examined.

The pathophysiology of PAH is characterized by increased pulmonary vascular resistance (PVR) at the beginning, followed by elevated pulmonary arterial pressure (PAP), decreased cardiac output, and increased RAP. The published data have supported that the hemodynamic indices, including PVR, cardiac output, and RAP were predictive of clinical outcomes among PAH patients [4, 5]. However, it remains debated whether the changes in the hemodynamic parameters are predictive of clinical outcomes. Although the non-invasive variables have been widely recommended to assess the risks in PAH, the mismatch between pulmonary resistance and RV contractility remains the main cause of mortality. We, therefore, conducted a systemic review to investigate the prognostic values of the changes in hemodynamic indices in PAH.

## Methods

The protocol for this review has been registered in PROSPERO (registration number CRD42019125157), and the study followed the Preferred Reporting Items for Systematic Reviews and Meta-Analyses (PRISMA) guidelines version 2020 (Supplementary Table 1) [6].

### Search strategy

All relevant studies from EMBASE, MEDLINE, Cochrane Library, and PubMed through August 2021, were searched and identified using the following keywords and the Medical Subject Headings (MeSH) terms: Pulmonary hypertension, Pulmonary Arterial Hypertension, PH, and PAH. No language restrictions were applied on any of these searches. We limited our searches to randomized controlled trials (RCTs) that compare either the effects of any of the 9 drug classes (ERA, PDE5, PDGFR, Prostacyclin, Prostacyclin plus ERA, Rho-kinase, TXSI/TXRA, sGC) with placebo or the effects between 2 drug classes. In the process of formulating the search strategy, the research team not only revised and discussed the preliminary search results to find a consensus but also consulted the librarians of the research institution to refer to their suggestions. Given the study is the secondary analysis of the published data, the review was waived by the ethical committee of Taipei Veterans General Hospital.

### Inclusion and exclusion criteria

**Fig. 1:**
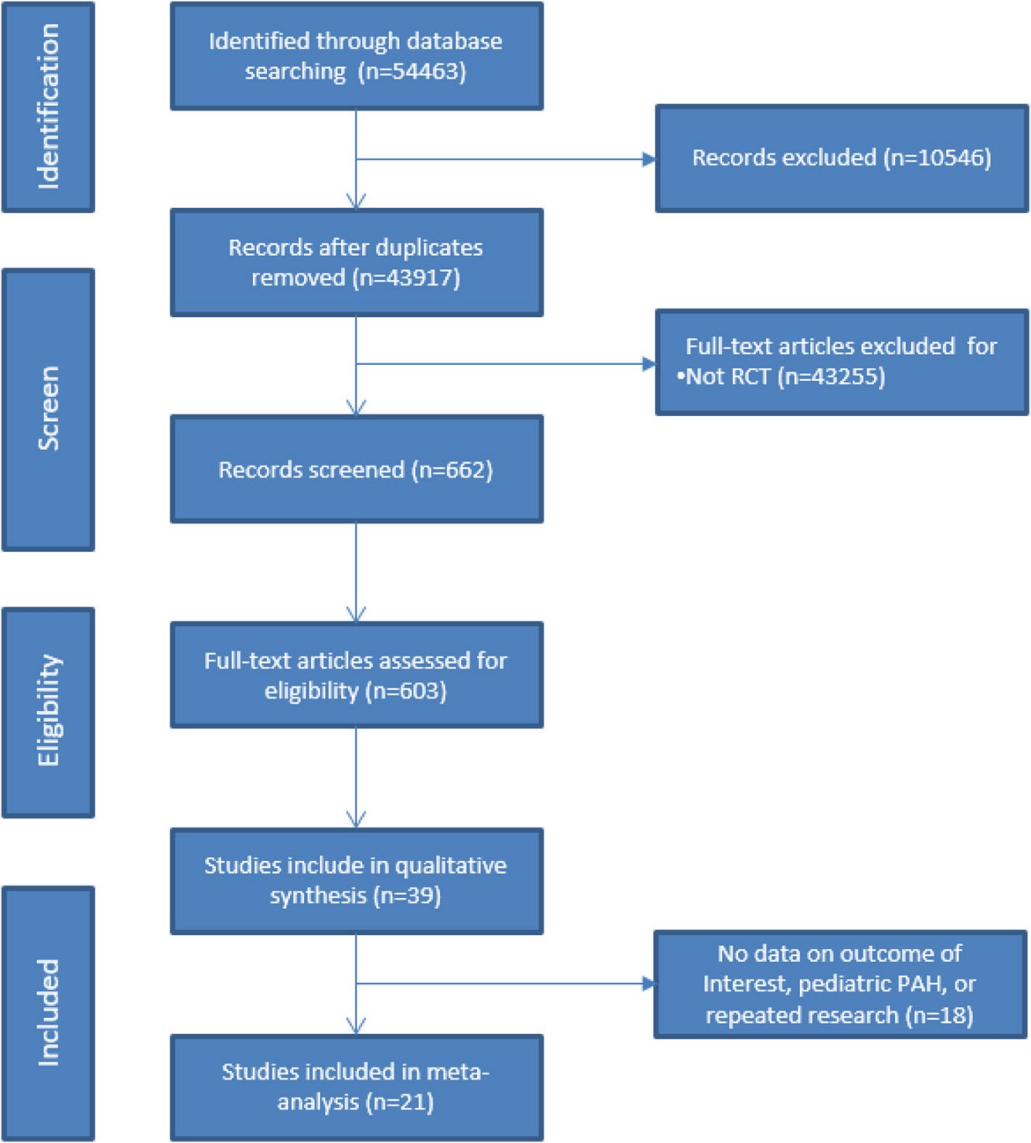
Flow chart of the literature search for studies investigating the effects of drugs on PAH including hemodynamic parameters. RCT stands for randomized controlled trials

Studies were eligible only if they reported any or all of the following outcomes: hospitalization for PAH, death due to PAH, total mortality, all adverse events of hospitalization and all-cause death, and exercise capacity (as measured by a 6-min walk distance, 6MWD). Additional studies were retrieved by manually checking the reference lists of reviews, meta-analyses, and original publications. Finally, we excluded RCT studies investigating pediatric PAH (age < 12) and those that did not report sequential measurements of cardiopulmonary hemodynamics, including mPAP, PVR, RAP, CI, or pulmonary artery wedge pressure (PAWP). For studies with more than one publication, only the studies with the largest number of participants in the trial were retained. The search of eligible studies was done separately by 2 investigators (W. Y. Yeh and W.M. Huang). The consensus was then reached through discussion and the arbitration of the principal investigator (H.M. Cheng). Of 603 articles identified by the initial search, 39 were retrieved for more detailed evaluation, and 21 trials were included in the study. The selection process of the literature search is shown in Fig. 1. The International prospective register of systematic reviews (PROSPERO) registration number of this study is CRD42019125157 (URL: https://www.crd.york.ac.uk/PROSPERO/).

### Data extraction

To calculate the unit consistency, the standard deviations were all converted to standard error (divided by the square root of the sample number), 95% confidence intervals were converted to standard errors (= [upper limit-lower limit]/3.92). If the actual data is not presented in the study and only graphically, we use WebPlotDigitizer version 4.1 [7] to interpolate the approximate data. In addition, for studies reporting PVR in Woods units, we multiplied this value by 80 to obtain the PVR in dyne-sec/cm^5^.

Data were extracted from papers by 2 investigators (W. Y. Yeh and C. J. Huang) independently, and differences in data extraction were resolved through discussions with the third investigator (H.M. Cheng).

### Data synthesis and statistical analysis

Weighted meta-regression analysis was performed to examine the relationship between hemodynamics changes before/after the interventions and outcome variables included in this study by Comprehensive Meta-Analysis version 3.3.070 [8]. For this analysis, the achieved differences between the changes in 6MWD (∆6MWD), and the event numbers of hospitalization and deaths in active treatment and control groups were considered.

For the assessment of the regression coefficient of each hemodynamic parameter with ∆6MWD and clinical outcomes, and changes in mPAP (∆mPAP), PVR (∆PVR), RAP (∆RAP), and CI (∆CI), were entered into the meta-regression model separately with the adjustment of age, sex, and baseline WHO function class. The prognostic values of ∆mPAP, ∆PVR, ∆RAP, ∆CI, and ∆6MWD were evaluated by using the univariate meta-regression model. For all meta-regression analyses, a random-effects model was used, and the analogous *R* square value (*R*^2^ analog) was adopted to quantify the proportion of variance explained by the entered covariate(s) in meta-regression. Tau^2^ and the restricted maximum likelihood (REML) methods were used to explain residual heterogeneity not explained by the covariate(s) [8, 9]. If there were missing values of the hemodynamic parameters or outcomes in the enrolled studies, the missing data were excluded from the meta-regression analysis.

### Assessment of risk of bias and level of evidence

The quality of studies was assessed by using the Cochrane Risk of Bias tool to assess the quality of these randomized controlled trials. The following 7 main domains are used in the assessment: (1) bias arising from the randomization; (2) bias due to inappropriate allocation process; (3) bias due to blinding of participants or outcome data assessment; (4) bias due to missing outcome data; (5) bias in measurement of the outcome; (6) bias in selection of the reported result; (7) other bias that may significantly affect the interpretation of the results. Bias is assessed as a judgment of high, low, or unclear. Trials with high or unclear risk for bias were considered with a high risk of bias. The quality of overall evidence and strength of recommendation for ∆6MWD (Y1), all adverse events (Y2), total mortality (Y3), hospitalization for PAH (Y4), and death due to PAH (Y5) were further determined (refer to the Grading of Recommendations, Assessment, Development and Evaluations (GRADE) approach with the use of GRADEpro software (https://gradepro.org/)) [10], including the dimensions of Indirectness, Imprecision, Publication bias, and the Certainty/Importance of the overall evidence, while the indicator of effect is modified to the adjusted regression coefficient of meta-regression (Supplemental Table S1).

Each item of the Cochrane Risk of Bias tool and Grade tool were also independently assessed by 2 investigators (S. H. Sung and W. Y. Yeh), and the disparities during this assessment process were determined by the principal investigator (H.M. Cheng).

## Results

### Characteristics of the included studies

A total of 21 RCTs and 3306 PAH patients, published between 1996 and 2013 were recruited in this analysis [11-31]. Supplemental Table S2 has shown the characteristics of each RCT, and the mean age of the study population ranged from 29 to 56 years. Of all the participants, 1097 received a placebo, and 2166 were treated with active drugs. The changes in hemodynamics indices, including ∆mPAP, ∆PVR, ∆RAP, and ∆CI, ∆6MWD, and the adverse events of mortality, death due to PAH, and hospitalization for PAH were summarized in Supplemental Table S3.

### Meta-regression of the hemodynamic parameters on clinical outcomes

**Fig. 2:**
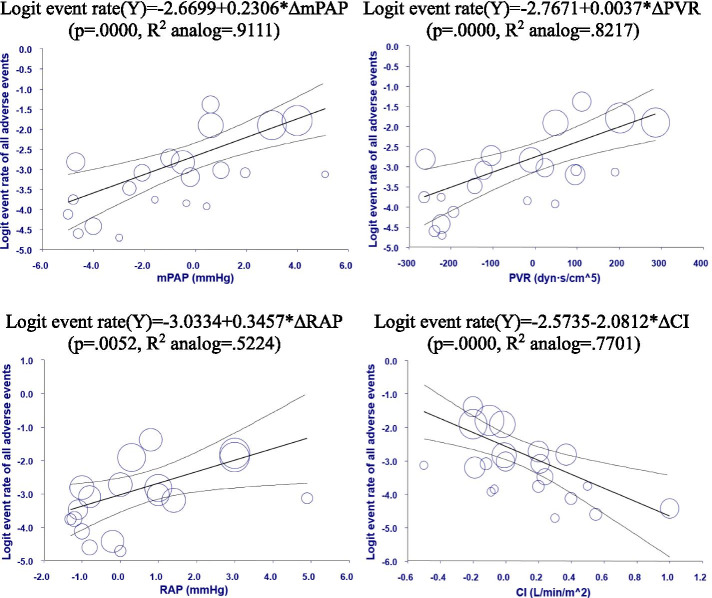
Univariate meta-regression analysis between changes in (1) mPAP, (2) PVR, (3) RAP, (4) CI, and all adverse events (Y). Only statistically significant relationships in table are plotted in this figure. mPAP, mean pulmonary artery pressure; PVR, pulmonary vascular resistance; RAP, right atrial pressure; CI, right ventricle cardiac output index. Each figure also shows the regression lines (bold straight lines) and their 95% confidence intervals (the thinner lines above and below the regression line represents the upper and lower limits). The size of the circles represents the importance of the study results in the regression estimates

The meta-regression analysis demonstrated that all of the changes in hemodynamic indices, including ∆mPAP, ∆PVR, ∆RAP, and ∆CI, correlated with the ∆6MWD, after accounting for age, sex, and baseline functional class (Table 1, Fig. 2). Patients with increasing mPAP, PVR, and RAP were independently associated with less improvement of 6MWD (*β* = −7.1067, −0.1046, and −10.6923, respectively), and increasing change in CI was independently related to better improvement of 6MWD (*β* = 42.4492). Concerning the clinical outcomes, after accounting for age, sex, and baseline functional class, increased ∆mPAP and ∆PVR, and decreased ∆CI were associated with more adverse clinical events (*β* = 0.1794, 0.0031, and −1.7544, respectively) (Table 1, Fig. 2). While ∆mPAP was the variable with the highest explanatory power for ∆6MWD (*R*^2^ analog = 0.74), it also had the highest explanatory power for the incident adverse events (*R*^2^ analog = 0.91) among the 4 hemodynamic indices. On the other hand, increased ∆PVR and decreased ∆CI were related to higher mortality rates (*β* = 0.0022 and −1.2136, respectively). In addition, none of the changes in hemodynamic indices was significantly related to the hospitalizations for PAH or death due to PAH in multivariate meta-regression analysis. Moreover, ∆6MWD did not correlate with any adverse event, neither.

**Table 1:** Meta-regression analysis of the relationship between the hemodynamic parameters and PAH prognostic outcomes

Outcomes	Predictors	Coefficient^#^	*P* value^#^	*R*^2^ analog^a^
(Y1) ∆6MWD	(X1) ∆mPAP	**−7.1067**	**< .0001**	**.7397**
	(X2) ∆PVR	**−0.1046**	**< .0001**	**.6442**
	(X3) ∆RAP	**−**10**.6923**	**.0093**	**.1011**
	(X4) ∆CI	**42.4492**	**< .0001**	**.4574**
(Y2) All adverse events	(X1) ∆mPAP	**0.1794**	**.0048**	**.9111**
	(X2) ∆PVR	**0.0031**	**.0015**	**.8217**
	(X3) ∆RAP	0.2191	.1109	.5224
	(X4) ∆CI	**−1.7544**	**.0004**	**.7701**
	(X5) ∆6MWD	**−**0.0141	.1195	.6511
(Y3) Total mortality	(X1) ∆mPAP	0.1143	.0932	.5498
	(X2) ∆PVR	**0.0022**	**.0332**	**.6116**
	(X3) ∆RAP	0.2412	.0796	.5567
	(X4) ∆CI	**−1.2136**	**.0250**	**.6055**
	(X5) ∆6MWD	**−**0.0080	.2600	.7046
(Y4) Hospitalization for PAH	(X1) ∆mPAP	0.1420	.0519	.2792
	(X2) ∆PVR	0.0024	.1174	.5761
	(X3) ∆RAP	0.0733	.6571	.0000
	(X4) ∆CI	**−**0.6668	.3520	.0000
	(X5) ∆6MWD	**−**0.0079	.2847	.0704
(Y5) Death due to PAH	(X1) mPAP	0.0548	.6496	.0000
	(X2) ∆PVR	0.0020	.4046	.0000
	(X3) ∆RAP	**−**0.0016	.9552	.0000
	(X4) ∆CI	**−**0.7086	.5299	.0000
	(X5) ∆6MWD	0.0005	.9688	.0000

^#^Adjusted for age, sex, and baseline WHO function class

^a^Results of univariate regression analysis

### Risks of bias among the included studies

The risk of bias among the 21 RCTs was evaluated in 7 categories (Supplemental Figure S1), and researchers presented higher or uncertain risk aspects including (1) allocation concealment: 18 articles had no detailed description; (2) blinding of outcome assessment: 15 articles had no clear description, but the 6MWD, hospitalizations, and mortality were objectively evaluated indicators, which were less susceptible to human subjective assessment; (3) selective reporting: 13 articles unlisted study protocol to check the reported and unreported findings, nor a special statement about the containment of all expected outcomes; therefore, there is a lack of sufficient information to determine whether it is possible to selectively report some study results. Because the main purpose of this study was to investigate the prognostic values of the changes in pulmonary hemodynamics, the study results should be less susceptible to the aforementioned risk of bias. Therefore, the 21 studies were all included in the subsequent meta-regression analysis.

### GRADE assessment

In addition, the assessment of the quality of the body of evidence was shown in Supplemental Table S4. In the GRADE Evidence Profile, the level of evidence for ∆6MWD was downgraded because of Indirectness concern (surrogate endpoint). Hospitalization for PAH was also downgraded because of Imprecision concerns (few incidents) (*n* = 4). Considering the overall quality of the evidence, together with the advantages and disadvantages of the clinical interventions for patients with PAH, consumption of medical resources, and patient values preferences, we considered ∆6MWD as “Important” for importance and rated other outcomes as “Critical important.”

## Discussion

In this meta-regression analysis of 21 RCTs and 3306 participants, we demonstrated all the changes in hemodynamic indices, including ∆mPAP, ∆PVR, ∆RAP, and ∆CI, were associated with ∆6MWD, independent of age, sex, and baseline functional class. However, only ∆mPAP, ∆PVR, and ∆CI, but not ∆RAP or ∆6MWD were related to clinical adverse events, after accounting for age, sex, and functional class. In addition, only ∆PVR and ∆CI were the hemodynamic parameters to be independently predictive of total mortality. But none of the changes in hemodynamic indices was correlated with PAH hospitalization or death due to PAH. The study results may support the use of the changes in the hemodynamic parameters, including ∆mPAP, for the risk assessment in the management of PAH.

### Hemodynamic indices and the prognosis

Although elevated mPAP is essential in the diagnosis of PAH and small increases in mPAP are independently associated with increased mortality in patients with borderline pulmonary hypertension [32, 33], several studies did not demonstrate any association between mPAP and the survival in PAH patients [4, 5]. Benza et al. have shown RAP but not mPAP was associated with 1-year survival in the REVEAL registry of 2716 subjects [34]. In contrast, CI among the hemodynamic indices was suggested to be predictive of clinical outcomes in European cohorts of PAH [3, 35]. Since the difference between mPAP and PAWP is the product of cardiac output multiplied by PVR, the increase in PVR and the decrease in cardiac output along with the progression of PAH could partially cancel their effects on mPAP. Conversely, an increase in mPAP could result from an augment in cardiac output due to improving PAH and might not be due to a rise in PVR from deteriorating PAH. Therefore, the determinants of mPAP could vary on different stages of right ventricular failure, and the prognostic value of mPAP in PAH patients is expected to be low. However, D’Alonzo et al. showed that a higher mPAP at the diagnosis of PAH conferred a greater risk of early death in a cohort of 194 patients [36]. Moreover, Sitbon et al. identified an paradoxical correlation between low baseline mPAP and mortality in 178 patients with PAH in WHO functional class III or IV [37]. In patients with severe PAH and right ventricular failure, low mPAP may better correlate with low cardiac output rather than low PVR, indicating worse outcomes [37]. On the other hand, few studies have investigated the association between the changes in hemodynamic indices and outcomes in patients with PAH. Weatherald et al. presented a PAH cohort of 981 patients who had undergone repeated hemodynamic surveys in a mean time of 4.6 months [38]. The results suggested that ∆mPAP and ∆PVR were significantly associated with death or lung transplantation in the whole study population, while ∆CI was only predictive of clinical outcomes in the subgroup of severe PAH patients [38].

In the present study, we have shown that ∆mPAP, ∆PVR, ∆RAP, and ∆CI were all crudely correlated with clinical adverse events. After accounting for age, sex, and WHO functional class, ∆mPAP, ∆PVR, and ∆CI remained significantly related to clinical outcomes. The study results may support the inclusion of these indices in the simplified risk score for the prediction of disease outcomes [35].

### The 6-min walk distance

The change from baseline in 6MWD (∆6MWD) has long-term served as the surrogate endpoint in the clinical trials of PAH to evaluate the therapeutic efficacy of the study drugs. It is expected that the indirect measure of 6MWD may reflect the clinically meaningful endpoints, such as quality of life and survival. The SERAPHIN study may have firstly endorsed the directly clinical outcomes as the primary endpoint to demonstrate that macitentan significantly reduced morbidity and mortality among patients with PAH [39]. However, the ∆6MWD was not associated with the long-term outcomes [40]. In a meta-analysis of 16 short-term RCTs, Macchia et al. have shown the ∆6MWD was not predictive of a survival benefit or adverse clinical events [41]. The updated meta-analyses have demonstrated again that the ∆6MWD did not correlate with any of the composite clinical events, including mortality, hospitalization for PAH, lung transplantation, or the initiation of rescue therapy [42, 43]. The present study also found that ∆6MWD was not associated with clinical outcomes. The results support the use of morbidity and mortality rather than ∆6MWD as the primary endpoint in the RCTs for PAH patients.

### Limitations

The long-term prognostic values of hemodynamic changes have not been evaluated in large cohorts yet. Although meta-regression analysis may improve our understandings of the associations between hemodynamic indices and the long-term clinical outcomes, the variances of baseline characteristics, study designs, and background therapies across the enrolled RCTs can cause biased study findings. Some RCTs were undertaken to prove the short-term effects of a novel drug mainly on exercise capacity. Although the others might have been designed to evaluate the therapeutic effects on long-term mortality and morbidities, caution should be exercised to interpret the correlations between hemodynamic changes and clinical outcomes. For patients with early PAH and preserved right ventricular function, the therapeutic changes in CI might be subtle, and the changes in mPAP may reflect the changes in PVR. In patients with PAH and profound right ventricular failure, improvement of CI followed by increased mPAP may indicate significant amelioration of right ventricular dysfunction, and better long-term outcomes were expected. While connective tissue disease is the second common etiologies of PAH, it may cause direct damage on the myocardium rather than through PAH. The inclusion of these subjects with distinct pathophysiology in the previously published RCTs may influence the findings observed in the present meta-regression analysis. In addition, lung transplantation was not statistically reported as an isolated endpoint in the majority of the studies. Although lung transplantation was even identical to the mortality event, the study was not able to analyze the associated impacts due to insufficient data. Moreover, the study results are based on published RCTs, in some of which currently available PAH drugs were not commercially available.

### Future directions

Given that the pulmonary hemodynamics are essentially related to long-term survival, the non-invasive assessments of the risk features are currently encouraged for the management of PAH to improve the guideline implementation [35]. While the cross-sectional hemodynamic evaluations have been predictive of clinical events, the measures of the changes in hemodynamics may further disclose the prognostic information in response to PAH therapy. However, future studies are needed to evaluate the effectiveness of hemodynamic-guided treatment, stratified by PAH etiologies and right ventricular function.

## Conclusions

Progression of PAH is usually characterized by increasing mPAP, PVR, and RAP, and decreasing CI, and this study aggregates the evidence of existing RCTs for verification. In addition to the baseline and on-treatment hemodynamic measures, the present study demonstrates that ∆mPAP, ∆PVR, ∆RAP, and ∆CI were all significantly associated with the surrogate endpoint (∆6MWD). Furthermore, ∆PVR and ∆CI were significantly associated with mortality, and ∆mPAP, ∆PVR, and ∆CI correlated with adverse clinical events of PAH patients. Given that risk stratification is essential in the management of PAH, further studies are warranted to evaluate whether the changes in the hemodynamic indices could be used to evaluate the therapeutic effects, in addition to the clinical risk factors, including functional class, 6MWD, and NT-proBNP.

## Supplementary Information

**Additional file 1: Supplemental Table S1.** The PRISMA 2020 checklist. **Supplemental Table S2.** Characteristics of the randomized controlled trials identified for the study (2-1). **Supplemental Table S3.** Characteristics of the randomized controlled trials identified for the study (2-2). **Supplemental Table S4.** GRADE Evidence Profile of Certainity assessment and Importance. **Supplemental Figure S1.** Risk of bias summary of 21 studies according to the Cochrane Handbook for Systematic Reviews of Interventions (“+”: low risk; “-”: high risk; “?”: unclear risk).

## Data Availability

The database of the present study can be made available on reasonable request to the corresponding author.

## Abbreviations

PAH: Pulmonary arterial hypertension
mPAP: Pulmonary artery pressure
PVR: Pulmonary vascular resistance
RAP: Right atrial pressure
CI: Cardiac output index
6WMD: 6-min walking distance
MeSH: Medical Subject Headings
