# Supplementary material for: The prognostic significance of the alterations of pulmonary hemodynamics in patients with pulmonary arterial hypertension: a meta-regression analysis of randomized controlled trials
Source: Syst Rev. 2021 Oct 30;10:284. doi: 10.1186/s13643-021-01816-0 (PMC8556931; doi:10.1186/s13643-021-01816-0)
Supplement: Supplementary file 1 — Additional file 1: Supplemental Table S1. The PRISMA 2020 checklist. Supplemental Table S2. Characteristics of the randomized controlled trials identified for the study (2-1). Supplemental Table S3. Characteristics of the randomized controlled trials identified for the study (2-2). Supplemental Table S4. GRADE Evidence Profile of Certainity assessment and Importance. Supplemental Figure S1. Risk of bias summary of 21 studies according to the Cochrane Handbook for Systematic Reviews of Interventions (“+”: low risk; “-”: high risk; “?”: unclear risk). [file 13643_2021_1816_MOESM1_ESM.docx]

**Supplemental Table S1.** The PRISMA 2020 checklist

| **Section and Topic** | **Item #** | **Checklist item** | **Location where item is reported** |
| --- | --- | --- | --- |
| **TITLE** | | |  |
| Title | 1 | Identify the report as a systematic review. | Title page |
| **ABSTRACT** | | |  |
| Abstract | 2 | See the PRISMA 2020 for Abstracts checklist. | 4-5 |
| **INTRODUCTION** | | |  |
| Rationale | 3 | Describe the rationale for the review in the context of existing knowledge. | 7 |
| Objectives | 4 | Provide an explicit statement of the objective(s) or question(s) the review addresses. | 8 |
| **METHODS** | | |  |
| Eligibility criteria | 5 | Specify the inclusion and exclusion criteria for the review and how studies were grouped for the syntheses. | 9 |
| Information sources | 6 | Specify all databases, registers, websites, organisations, reference lists and other sources searched or consulted to identify studies. Specify the date when each source was last searched or consulted. | 8-9 |
| Search strategy | 7 | Present the full search strategies for all databases, registers and websites, including any filters and limits used. | 8-9 |
| Selection process | 8 | Specify the methods used to decide whether a study met the inclusion criteria of the review, including how many reviewers screened each record and each report retrieved, whether they worked independently, and if applicable, details of automation tools used in the process. | 8-9 |
| Data collection process | 9 | Specify the methods used to collect data from reports, including how many reviewers collected data from each report, whether they worked independently, any processes for obtaining or confirming data from study investigators, and if applicable, details of automation tools used in the process. | 9-10 |
| Data items | 10a | List and define all outcomes for which data were sought. Specify whether all results that were compatible with each outcome domain in each study were sought (e.g. for all measures, time points, analyses), and if not, the methods used to decide which results to collect. | 10-11 |
|  | 10b | List and define all other variables for which data were sought (e.g. participant and intervention characteristics, funding sources). Describe any assumptions made about any missing or unclear information. | 9-10 |
| Study risk of bias assessment | 11 | Specify the methods used to assess risk of bias in the included studies, including details of the tool(s) used, how many reviewers assessed each study and whether they worked independently, and if applicable, details of automation tools used in the process. | 11-12 |
| Effect measures | 12 | Specify for each outcome the effect measure(s) (e.g. risk ratio, mean difference) used in the synthesis or presentation of results. | 10-11 |
| Synthesis methods | 13a | Describe the processes used to decide which studies were eligible for each synthesis (e.g. tabulating the study intervention characteristics and comparing against the planned groups for each synthesis (item #5)). | Supplemental Table S2  Figure 1 |
|  | 13b | Describe any methods required to prepare the data for presentation or synthesis, such as handling of missing summary statistics, or data conversions. | 9-11 |
|  | 13c | Describe any methods used to tabulate or visually display results of individual studies and syntheses. | 9-11 |
|  | 13d | Describe any methods used to synthesize results and provide a rationale for the choice(s). If meta-analysis was performed, describe the model(s), method(s) to identify the presence and extent of statistical heterogeneity, and software package(s) used. | 9-11 |
|  | 13e | Describe any methods used to explore possible causes of heterogeneity among study results (e.g. subgroup analysis, meta-regression). | 10-11 |
|  | 13f | Describe any sensitivity analyses conducted to assess robustness of the synthesized results. | N/A |
| Reporting bias assessment | 14 | Describe any methods used to assess risk of bias due to missing results in a synthesis (arising from reporting biases). | 11-12 |
| Certainty assessment | 15 | Describe any methods used to assess certainty (or confidence) in the body of evidence for an outcome. | 11-12 |
| **RESULTS** | | |  |
| Study selection | 16a | Describe the results of the search and selection process, from the number of records identified in the search to the number of studies included in the review, ideally using a flow diagram. | 12  Figure 1 |
|  | 16b | Cite studies that might appear to meet the inclusion criteria, but which were excluded, and explain why they were excluded. | N/A |
| Study characteristics | 17 | Cite each included study and present its characteristics. | Supplemental Table S2  Supplemental Table S3 |
| Risk of bias in studies | 18 | Present assessments of risk of bias for each included study. | Supplemental Figure S1 |
| Results of individual studies | 19 | For all outcomes, present, for each study: (a) summary statistics for each group (where appropriate) and (b) an effect estimate and its precision (e.g. confidence/credible interval), ideally using structured tables or plots. | Table 1  Figure 2  Supplemental Table S4 |
| Results of syntheses | 20a | For each synthesis, briefly summarise the characteristics and risk of bias among contributing studies. | 13 |
|  | 20b | Present results of all statistical syntheses conducted. If meta-analysis was done, present for each the summary estimate and its precision (e.g. confidence/credible interval) and measures of statistical heterogeneity. If comparing groups, describe the direction of the effect. | Table 1  Figure 2  Supplemental Table S4 |
|  | 20c | Present results of all investigations of possible causes of heterogeneity among study results. | N/A |
|  | 20d | Present results of all sensitivity analyses conducted to assess the robustness of the synthesized results. | N/A |
| Reporting biases | 21 | Present assessments of risk of bias due to missing results (arising from reporting biases) for each synthesis assessed. | Supplemental Table S4 |
| Certainty of evidence | 22 | Present assessments of certainty (or confidence) in the body of evidence for each outcome assessed. | Supplemental Table S4 |
| **DISCUSSION** | | |  |
| Discussion | 23a | Provide a general interpretation of the results in the context of other evidence. | 14-16 |
|  | 23b | Discuss any limitations of the evidence included in the review. | 16-17 |
|  | 23c | Discuss any limitations of the review processes used. | 16-17 |
|  | 23d | Discuss implications of the results for practice, policy, and future research. | 18 |
| **OTHER INFORMATION** | | |  |
| Registration and protocol | 24a | Provide registration information for the review, including register name and registration number, or state that the review was not registered. | 5  8 |
|  | 24b | Indicate where the review protocol can be accessed, or state that a protocol was not prepared. | 5  8 |
|  | 24c | Describe and explain any amendments to information provided at registration or in the protocol. | N/A |
| Support | 25 | Describe sources of financial or non-financial support for the review, and the role of the funders or sponsors in the review. | 2 |
| Competing interests | 26 | Declare any competing interests of review authors. | 2 |
| Availability of data, code and other materials | 27 | Report which of the following are publicly available and where they can be found: template data collection forms; data extracted from included studies; data used for all analyses; analytic code; any other materials used in the review. | Supplemental Table S2  Supplemental Table S3 |

*From:*  Page MJ, McKenzie JE, Bossuyt PM, Boutron I, Hoffmann TC, Mulrow CD, et al. The PRISMA 2020 statement: an updated guideline for reporting systematic reviews. BMJ 2021;372:n71. doi: 10.1136/bmj.n71

For more information, visit: <http://www.prisma-statement.org/>

**Supplemental Table S2.** Characteristics of the randomized controlled trials identified for the study (2-1)

| **Study/Ref No** | **Study** | **Trial acronym** | **Year** | **PAH Drug Class** | **PAH drugs** | **Brand name of PAH drugs** | **Treatment group population, n** | **Follow-up durations** | **Mean or median age (y)** | **# of female (%)** | **PAH causes, n** | | **WHO baseline functional class, case number** | | | |
| --- | --- | --- | --- | --- | --- | --- | --- | --- | --- | --- | --- | --- | --- | --- | --- | --- |
|  |  |  |  |  |  |  |  |  |  |  | **primary** | **associated** | **I** | **II** | **III** | **IV** |
| 1 | Barst 1996 |  | 1996 | control | conventional therapy |  | 40 | 12 weeks | 40 | 28 (70) | 40 |  | 0 | 0 | 29 | 11 |
| 1 |  |  | 1996 | epoprostenol | epoprostenol (prostacyclin or prostaglandin I_2_)+  conventional therapy | initial rate of 2 ng/kg  of body weight per minute, with increments of 2 ng/kg  per minute every 15 minutes | 41 | 12 weeks | 40 | 31 (76) | 41 |  | 0 | 0 | 31 | 10 |
| 2 | Channick 2001 |  | 2001 | Placebo | Placebo | - | 11 | 12 weeks | 47.4 | 11 (100) | 10 | 1 | 0 | 0 | 11 | 0 |
| 2 |  |  | 2001 | ERA | ERA | bosentan (62.5mg, twice daily x4weeks, then 125mg twice daily) | 21 | 12 weeks | 52.2 | 17 (81) | 17 | 4 | 0 | 0 | 21 | 0 |
| 3 | Galie` 2002 | ALPHABET | 2002 | Placebo | Control | - | 65 | 12 weeks | 45.1 | 38 (58.5) | 28 | 37 | 0 | 33 | 32 | 0 |
| 3 |  | ALPHABET | 2002 | Prostacyclin | IV prostacyclin | beraprost, median dose 80microgram four times a day | 65 | 12 weeks | 45.8 | 42 (64.6) | 35 | 30 | 0 | 31 | 34 | 0 |
| 4 | Olschewski 2002 |  | 2002 | Placebo | Control | - | 102 | 12 weeks | 52.8 | 68 (66.7) | 51 | 51 | 0 | 0 | 59 | 43 |
| 4 |  |  | 2002 | Prostacyclin | IH prostacyclin | iloprost, 2.5 or 5.0μg, six or nine times/day | 101 | 12 weeks | 51.2 | 69 (68.3) | 51 | 50 | 0 | 0 | 60 | 41 |
| 5 | Langleben 2002 |  | 2002 | Placebo | Control | - | 25 | 12 weeks | 46.3 | 23 (92) |  |  | 0 | 10 | 15 | 0 |
| 5 |  |  | 2002 | TXSI/TXRA | TXSI/TXRA | terbogrel,100 mg twice daily | 23 | 12 weeks | 45.3 | 17 (73.9) |  |  | 0 | 14 | 9 | 0 |
| 5 |  |  | 2002 |  | TXSI/TXRA | Terbogrel,200 mg twice daily | 23 | 12 weeks | 49.3 | 15 (65.2) |  |  | 0 | 11 | 12 | 0 |
| 6 | Simonneau 2002 |  | 2002 | Placebo | Control | - | 236 | 12 weeks | 44.4 | 185 (78.4) | 136 | 100 | 0 | 28 | 192 | 16 |
| 6 |  |  | 2002 | Prostacyclin | SC prostacyclin | treprostinil, 1.25 ng/kg/min to maximum dose | 233 | 12 weeks | 44.6 | 197 (84.5) | 134 | 99 | 0 | 25 | 190 | 18 |
| 7 | Barst 2003 |  | 2003 | Placebo | Control | - | 56 | 12 months | 42 | 47 (83.9) | 40 | 16 | 0 | 28 | 28 | 0 |
| 7 |  |  | 2003 | Prostacyclin | oral prostacyclin analogue | beraprost sodium, median dose 120 g four times a day | 60 | 12 months | 42 | 52 (86.7) | 47 | 13 | 0 | 33 | 27 | 0 |
| 8 | Barst 2004 | STRIDE 1 | 2004 | Placebo | Control | - | 60 | 12 weeks | 48 | 47 (78.3) | 37 | 23 | 0 | 22 | 36 | 2 |
| 8 |  | STRIDE 1 | 2004 | ERA | ERA | sitaxsentan, 100mg orally once daily | 55 | 12 weeks | 45 | 47 (85.5) | 23 | 32 | 0 | 16 | 39 | 0 |
| 8 |  | STRIDE 1 | 2004 |  | ERA | sitaxsentan, 300mg orally once daily | 63 | 12 weeks | 44 | 47 (74.6) | 34 | 29 | 0 | 21 | 42 | 0 |
| 9 | Galiè 2005 | SUPER | 2005 | Placebo | Control | - | 70 | 12 weeks | 49 | 57 (81.4) | 42 | 28 | 1 | 32 | 34 | 3 |
| 9 |  | SUPER | 2005 | PDE5 inhibitor | PDE5 inhibitor | sildenafil, 20mg, orally three times daily | 69 | 12 weeks | 47 | 49 (71) | 44 | 25 | 0 | 24 | 40 | 5 |
| 9 |  | SUPER | 2005 |  | PDE5 inhibitor | sildenafil, 40mg, orally three times daily | 67 | 12 weeks | 51 | 47 (70.1) | 43 | 24 | 0 | 23 | 44 | 0 |
| 9 |  | SUPER | 2005 |  | PDE5 inhibitor | sildenafil, 80mg, orally three times daily | 71 | 12 weeks | 48 | 56 (78.9) | 46 | 25 | 0 | 28 | 42 | 1 |
| 10 | Wilkins 2005 | SERAPH | 2005 | PDE5 inhibitor | PDE5 inhibitor | sildenafil, 50 mg twice daily for 4 weeks, then 50 mg three times daily | 14 | 16 weeks | 44.4 | 11 (78.6) | 12 | 2 |  |  |  |  |
| 10 |  | SERAPH | 2005 | ERA | ERA | control group: bosentan (62.5 mg twice daily for 4 weeks, then 125 mg twice daily) | 12 | 16 weeks | 41.1 | 10 (83.3) | 11 | 1 |  |  |  |  |
| 11 | Galiè 2006 | BREATHE-5 | 2006 | Placebo | Control | - | 17 | 16 weeks | 44.2 | 10 (58.8) | 0 | 17 | 0 | 0 | 17 | 0 |
| 11 |  | BREATHE-5 | 2006 | ERA | ERA | bosentan 62.5 mg BID x4 weeks, then 125mg BID x12 weeks | 37 | 16 weeks | 37.2 | 23 (62.2) | 0 | 37 | 0 | 0 | 37 | 0 |
| 12 | McLaughlin 2006 |  | 2006 | ERA | ERA | bosentan (pre-inclusion) | 33 | 12 weeks | 49 | 26 (78.8) | 20 | 13 | 0 | 1 | 30 | 2 |
| 12 |  |  | 2006 | Prostacyclin plus ERA | IH prostacyclin plus ERA | inhaled iloprost (5 mug) plus bosentan (pre-inclusion) | 34 | 12 weeks | 51 | 27 (79.4) | 17 | 17 | 0 | 0 | 35 | 1 |
| 13 | Badesch 2007 | SUPER-1 | 2007 | Placebo | Control | - | 22 | 12 weeks | 56 | 18 (81.8) | 0 | 22 | 0 | 9 | 13 | 0 |
| 13 |  | SUPER-1 | 2007 | PDE5 inhibitor | PDE5 inhibitor | sildenafil, 20mg, orally three times daily | 21 | 12 weeks | 52 | 16 (76.2) | 0 | 21 | 0 | 7 | 13 | 1 |
| 13 |  | SUPER-1 | 2007 |  | PDE5 inhibitor | sildenafil, 40mg, orally three times daily | 20 | 12 weeks | 50 | 17 (85) | 0 | 20 | 0 | 7 | 13 | 0 |
| 13 |  | SUPER-1 | 2007 |  | PDE5 inhibitor | sildenafil, 80mg, orally three times daily | 21 | 12 weeks | 54 | 19 (90.5) | 0 | 21 | 0 | 9 | 12 | 0 |
| 14 | Simonneau 2008 |  | 2008 | Prostacyclin | Intravenous prostacyclin | Intravenous epoprostenol | 133 | 16 weeks | 47.5 | 103 (77.4) | 105 | 28 | 2 | 34 | 87 | 6 |
| 14 |  |  | 2008 | Prostacyclin plus PDE5 inhibitor | Intravenous prostacyclin plus PDE5I | Intravenous epoprostenol plus Sildenafil | 134 | 16 weeks | 47.8 | 110 (82.1) | 107 | 27 | 1 | 34 | 88 | 10 |
| 15 | Galiè 2008 | EARLY | 2008 | Placebo | Control | - | 92 | 6 months | 44.2 | 58 (63) | 58 | 34 | 0 | 92 | 0 | 0 |
| 15 |  | EARLY | 2008 | ERA | ERA | bosentan | 93 | 6 months | 45.2 | 71 (76.3) | 54 | 39 | 0 | 93 | 0 | 0 |
| 16 | Galiè 2009 |  | 2009 | Placebo | Control | - | 82 | 16 weeks | 55 | 65 (79.3) | 54 | 28 | 1 | 23 | 56 | 2 |
| 16 |  |  | 2009 | PDE5 inhibitor | PDE5 inhibitor | tadalafil, 2.5mg | 82 | 16 weeks | 54 | 64 (78) | 45 | 37 | 1 | 29 | 49 | 3 |
| 16 |  |  | 2009 |  | PDE5 inhibitor | tadalafil, 10mg | 80 | 16 weeks | 54 | 67 (83.8) | 52 | 28 | 0 | 24 | 54 | 2 |
| 16 |  |  | 2009 |  | PDE5 inhibitor | tadalafil, 20mg | 82 | 16 weeks | 53 | 62 (75.6) | 50 | 32 | 0 | 28 | 54 | 0 |
| 16 |  |  | 2009 |  | PDE5 inhibitor | tadalafil, 40mg | 79 | 16 weeks | 53 | 59 (74.7) | 46 | 33 | 2 | 26 | 51 | 0 |
| 17 | Jing 2011 | EVALUATION | 2011 | Placebo | Placebo | - | 20 | 12 weeks | 29 | 17 (85) | 14 | 6 | 0 | 9 | 11 | 0 |
| 17 |  | EVALUATION | 2011 | PDE5 inhibitor | PDE5I | vardenafil (5 mg once daily for 4wk then 5mg twice daily | 44 | 12 weeks | 32 | 36 (81.8) | 25 | 19 | 0 | 21 | 23 | 0 |
| 18 | Zeng 2012 | APATH | 2012 | Placebo | Placebo | - | 108 | 6 months | 37 | 65 (60.2) | 34 | 74 | 0 | 71 | 37 | 0 |
| 18 |  | APATH | 2012 | Statin | statin | atorvastatin 10 mg daily | 112 | 6 months | 35 | 79 (70.5) | 30 | 82 | 0 | 70 | 42 | 0 |
| 19 | Fukumoto 2013 |  | 2013 | Placebo | placebo | - | 12 | 12 weeks | 51.4 | 10 (83.3) | 6 | 6 | 2 | 9 | 1 | 0 |
| 19 |  |  | 2013 | Rho-kinase inhibitor | Rho-kinase inhibitor | AT-877ER | 11 | 12 weeks | 47.4 | 6 (54.5) | 2 | 9 | 0 | 9 | 2 | 0 |
| 20 | Ghofrani 2013 | PATENT-1 | 2013 | Placebo | placebo | - | 126 | 12 weeks | 51 | 98 (77.8) | 84 | 42 | 4 | 60 | 58 | 3 |
| 20 |  | PATENT-1 | 2013 | sGC stimulator | soluble guanylate cyclase stimulator | riociguat, up to 2.5 mg three times daily | 254 | 12 weeks | 51 | 203 (79.9) | 149 | 105 | 5 | 108 | 140 | 1 |
| 20 |  | PATENT-1 | 2013 |  | soluble guanylate cyclase stimulator | riociguat, up to 1.5 mg three times daily | 63 | 12 weeks | 49 | 49 (77.8) | 39 | 24 | 5 | 19 | 39 | 0 |
| 21 | Simonneau 2012 |  | 2012 | Placebo | placebo |  | 10 | 17 weeks | 53.8 | 8 (80.0) | 8 | 2 | 0 | 2 | 8 | 0 |
| 21 |  |  | 2012 | Prostacyclin receptor | Selexipag | up-titrated in 200 μg increments from 200 μg  twice daily on Day 1 to maximum tolerated dose by Day 35 (maximum allowed dose of  800 μg twice daily) | 33 | 17 weeks | 54.8 | 27 (81.8) | 27 | 6 | 0 | 15 | 18 | 0 |

**Supplemental Table S3.** Characteristics of the randomized controlled trials identified for the study (2-2)

| **Study /Ref No** | **Study** | **PAH drugs** | **X1: ∆mPAP**  **(mmHg)** | **X2: ∆PVR (dyn·s/cm^5)** | **X3: ∆RAP**  **(mmHg)** | **X4: ∆CI**  **(L/min/m^2)** | **Y1: Delta 6MWD (m)** | | **Y2: All adverse events of hospitalization and death** | **Y3: Total mortality** | **Y4: Hospitalization for PAH** | **Y5: Death due to PAH** |
| --- | --- | --- | --- | --- | --- | --- | --- | --- | --- | --- | --- | --- |
|  |  |  | **MEAN** | **MEAN** | **MEAN** | **MEAN** | **MEAN** | **SE** | **# of Events** | **# of Events** | **# of Events** | **# of Events** |
| 1 | Barst 1996 | conventional therapy | -1.9 | -120 | 0.1 | -0.2 | -15 | 23.28 |  | 8 |  |  |
| 1 |  | epoprostenol (prostacyclin or prostaglandin I_2_)+  conventional therapy | -4.8 | -272 | -2.2 | 0.3 | 32 | 17.74 |  | 0 |  |  |
| 2 | Channick 2001 | Placebo | 5.1 | 191 | 4.9 | -0.5 | -6.00 | 93.80 | 0 | 0 | 0 | 0 |
| 2 |  | ERA | -1.6 | -223 | -1.3 | 0.5 | 70.00 | 52.50 | 0 | 0 | 0 | 0 |
| 3 | Galie` 2002 | Control | 1 | 24 | 1 | 0 |  |  | 3 | 2 | 0 | 1 |
| 3 |  | IV prostacyclin | -1 | -104 | 0 | 0.2 |  |  | 4 | 3 | 1 | 1 |
| 4 | Olschewski 2002 | Control | -0.2 | 96 | 1.4 | -0.19 |  |  | 4 | - | - | 4 |
| 4 |  | IH prostacyclin | -4.6 | -239 | -0.8 | 0.55 |  |  | 1 | - | - | 1 |
| 5 | Langleben 2002 | Control | 0.44 | 48 |  | -0.09 | -5.00 | 13.50 | 0 |  | 0 | 0 |
| 5 |  | TXSI/TXRA | -0.36 | -17.6 |  | -0.07 | 13.00 | 14.50 | 0 |  | 0 | 0 |
| 5 |  | TXSI/TXRA | 1.95 | 98.4 |  | -0.12 | 16.00 | 15.40 | 1 |  | 1 | 1 |
| 6 | Simonneau 2002 | Control | 0.7 | 96 | 1.4 | -0.06 |  |  | 12 | 0 | - | 7 |
| 6 |  | SC prostacyclin | -2.3 | -280 | -0.5 | 0.12 |  |  | 12 | 0 | - | 7 |
| 7 | Barst 2003 | Control | 2 |  | 1 | 0.1 |  |  | 9 | 0 |  | 2 |
| 7 |  | oral prostacyclin analogue | 1 |  | 0 | 0.1 |  |  | 6 | 0 |  | 1 |
| 8 | Barst 2004 | Control | 0 | 49 | 1 | 0 | -13.00 | 10.00 |  |  |  |  |
| 8 |  | ERA | -3 | -221 | 0 | 0.3 | 22.00 | 8.00 | 0 |  | 0 | 0 |
| 8 |  | ERA | -5 | -194 | -1 | 0.4 | 20.00 | 10.00 | 1 |  | 1 | 1 |
| 9 | Galiè 2005 | Control | 0.6 | 49 | 0.3 | -0.02 |  |  | 9 | 7 |  | 1 |
| 9 |  | PDE5 inhibitor | -2.1 | -122 | -0.8 | 0.21 | 45.00 | 6.25 | 3 | 2 |  | 1 |
| 9 |  | PDE5 inhibitor | -2.6 | -143 | -1.1 | 0.24 | 46.00 | 6.63 | 2 | 2 |  | 0 |
| 9 |  | PDE5 inhibitor | -4.7 | -261 | -1 | 0.37 | 50.00 | 6.89 | 4 | 2 |  | 2 |
| 10 | Wilkins 2005 | PDE5 inhibitor |  |  |  | 0.3 | 114.00 |  | 2 | 0 | 1 | 1 |
| 10 |  | ERA |  |  |  | 0.3 | 59.00 |  | 3 | 3 | 0 | 0 |
| 11 | Galiè 2006 | Control | 0.5 | 155.1 | 0.4 | -0.2 | -9.70 | 22.30 |  |  |  |  |
| 11 |  | ERA | -5 | -316.9 | 0.3 | 0.9 | 43.40 | 8.10 |  |  |  |  |
| 12 | McLaughlin 2006 | ERA | 2 | 81 |  | 0.1 | 4.00 | 61.00 |  | 4 |  |  |
| 12 |  | IH prostacyclin plus ERA | -6 | -164 |  | 0.1 | 30.00 | 60.00 |  |  |  |  |
| 13 | Badesch 2007 | Control | 1.4 | -19 | 0.4 | 0.08 | -13.00 | 11.73 |  |  | 0 | 0 |
| 13 |  | PDE5 inhibitor | -4.6 | -243 | -0.7 | 0.8 | 42.00 | 11.22 |  |  | 0 | 1 |
| 13 |  | PDE5 inhibitor | -2.8 | -144 | 0.2 | 0.4 | 36.00 | 11.22 |  |  | 0 | 0 |
| 13 |  | PDE5 inhibitor | -3.2 | -156 | 0.03 | 0.2 | 15.00 | 19.90 |  |  | 0 | 2 |
| 14 | Simonneau 2008 | Intravenous prostacyclin | 1.1 | 22.1 |  | -0.2 | 10.00 | 6.07 |  | 11 |  | 7 |
| 14 |  | Intravenous prostacyclin plus PDE5I | -2.8 | -150.6 |  | 0.6 | 29.80 | 5.79 |  | 8 |  | 0 |
| 15 | Galiè 2008 | Control | 3 |  | 1.1 | -0.15 | -7.90 | 8.20 |  | 3 | 0 | 1 |
| 15 |  | ERA | -2.7 |  | 0.5 | 0.09 | 11.20 | 7.90 |  | 1 | 0 | 1 |
| 16 | Galiè 2009 | Control |  |  |  |  |  |  |  |  |  | 1 |
| 16 |  | PDE5 inhibitor |  |  |  |  | 14.00 |  |  |  |  | 0 |
| 16 |  | PDE5 inhibitor |  |  |  |  | 20.00 |  |  |  |  | 1 |
| 16 |  | PDE5 inhibitor |  | -254 |  |  | 27.00 |  |  |  |  | 1 |
| 16 |  | PDE5 inhibitor |  | -209 |  |  | 33.00 |  |  |  |  | 0 |
| 17 | Jing 2011 | Placebo | 0.6 | 112 | 0.8 | -0.2 | -10.00 |  | 4 | 2 |  | 2 |
| 17 |  | PDE5I | -4.8 | -264 | -1.2 | 0.2 | 59.00 |  | 1 | 1 |  | 0 |
| 18 | Zeng 2012 | Placebo | 3 | 287 | 3 | -0.2 | -14.10 |  | 14 | 3 |  | 11 |
| 18 |  | statin | 4 | 203 | 3 | -0.1 | -16.60 | 18.11 | 16 | 7 |  | 9 |
| 19 | Fukumoto 2013 | placebo | 2.2 | 72.2 |  | 0.09 | 31.30 | 13.83 |  |  |  | 0 |
| 19 |  | Rho-kinase inhibitor | -0.6 | -31.8 |  | 0.368 | 18.90 | 9.74 |  |  |  | 1 |
| 20 | Ghofrani 2013 | placebo | -0.5 | -9 | 1 | -0.01 | -6.00 | 7.66 | 7 | 4 |  | 3 |
| 20 |  | soluble guanylate cyclase stimulator | -4 | -223 | -0.2 | 1 | 30.00 | 4.14 | 3 | 1 |  | 2 |
| 20 |  | soluble guanylate cyclase stimulator | -4 | -168 |  | 0.4 | 31.00 | 9.95 |  |  |  |  |
| 21 | Simonneau 2012 | placebo |  | 223.6 | -2.9 | -0.2 | 0.4 |  |  | 0 |  | 0 |
| 21 |  | Prostacyclin receptor |  | -129.8 | 0.3 | 0.3 | 24.6 |  |  | 0 |  | 0 |

mPAP: mean pulmonary artery pressure, PVR: pulmonary vascular resistance, RAP: Right atrial pressure, CI: right ventricle cardiac output index; 6MWD: change in 6-min walk distance (meters); All Adverse Events: the total events of hospitalization for PAH and death.

**Supplemental Table S4**. GRADE Evidence Profile of Certainity assessment and Importance

| **№ of studies** | **Certainty assessment** | | | | | | **Effect** | | | **Certainty** | **Importance** |
| --- | --- | --- | --- | --- | --- | --- | --- | --- | --- | --- | --- |
|  | **Study design** | **Risk of bias** | **Inconsistency** | **Indirectness** | **Imprecision** | **Publication bias** | **№ of events** | **№ of individuals** | **Regression**  **coefficient  (95% CI)** |  |  |
| (Y1) ∆6MWD | | | | | | | | | | | |
| 17 | randomised trials | not serious | not serious | serious | not serious | none | - | 2520 | (X1) ∆mPAP: -7.11 (-9.32~-4.90 )  (X2) ∆PVR: -0.10 (-0.14~ -0.07)  (X3) ∆RAP: -10.69 (-18.75~-2.63)  (X4) ∆CI: 42.45 (24.09~60.81) | ⨁⨁⨁◯ MODERATE | IMPORTANT |
| (Y2) All adverse events | | | | | | | | | | | |
| 12 | randomised trials | not serious | not serious | not serious | not serious | none | 121 | 2169 | (X1) ∆mPAP: 0.18 (0.05 ~0.30)  (X2) ∆PVR: 0.00 (0.00~0.01 )  (X3) ∆RAP: 0.22 (-0.05~0.49)  (X4) ∆CI: -1.75 (-2.72~-0.79)  (X5) ∆6MWD: -0.01 (-0.03~0.00) | ⨁⨁⨁⨁ HIGH | CRITCAL |
| (Y3) Total mortality | | | | | | | | | | | |
| 14 | randomised trials | not serious | not serious | not serious | not serious | none | 74 | 2323 | (X1) ∆mPAP: 0.11 (-0.02~0.25)  (X2) ∆PVR: 0.00 (0.00~0.00)  (X3) ∆RAP: 0.24 (-0.03~0.51)  (X4) ∆CI: -1.21 (-2.27~-0.15)  (X5) ∆6MWD: -0.01 (-0.02~0.01) | ⨁⨁⨁⨁ HIGH | CRITCAL |
| (Y4) Hospitalization for PAH | | | | | | | | | | | |
| 7 | randomised trials | not serious | not serious | not serious | serious | none | 4 | 646 | (X1) ∆mPAP: 0.14 (0.02~0.29)  (X2) ∆PVR: 0.00 (0.00~0.01)  (X3) ∆RAP: 0.07 (-0.25~0.40)  (X4) ∆CI -0.67 (-2.01~0.67)  (X5) ∆6MWD: -0.01 (-0.02~0.01) | ⨁⨁⨁◯ MODERATE | CRITCAL |
| (Y5) Death due to PAH | | | | | | | | | | | |
| 18 | randomised trials | not serious | not serious | not serious | not serious | none | 74 | 3113 | (X1) ∆mPAP: 0.05 (-0.18~0.29)  (X2) ∆PVR: 0.00 (0.00~0.01)  (X3) ∆RAP: 0.00 (-0.53~0.53)  (X4) ∆CI: -0.71 (-2.92~1.50)  (X5) ∆6MWD: 0.00 (-0.03~0.03) | ⨁⨁⨁⨁ HIGH | CRITCAL |


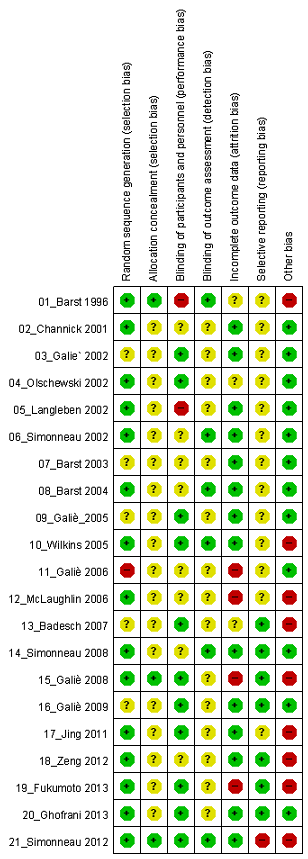


**Supplemental Figure S1**. Risk of bias summary of 21 studies according to the Cochrane Handbook for Systematic Reviews of Interventions (“+”: low risk; “-”: high risk; “?”: unclear risk)

**References in the Supplementary Material**

1. Barst RJ, Rubin LJ, Long WA, McGoon MD, Rich S, Badesch DB, et al. A comparison of continuous intravenous epoprostenol (prostacyclin) with conventional therapy for primary pulmonary hypertension. N Engl J Med. 1996;334(5):296–301.
2. Channick RN, Simonneau G, Sitbon O, Robbins IM, Frost A, Tapson VF, et al. Effects of the dual endothelin-receptor antagonist bosentan in patients with pulmonary hypertension: a randomised placebocontrolled study. Lancet. 2001;358(9288):1119–23.
3. Galie N, Humbert M, Vachiery J-L, Vizza C, Kneussl M, Manes A, et al. Effects of beraprost sodium, an oral prostacyclin analogue, in patients with pulmonary arterial hypertension: a randomized, double-blind, placebo-controlled trial. J Am Coll Cardiol. 2002;39(9):1496–502.
4. Olschewski H, Simonneau G, Galie N, Higenbottam T, Naeije R, Rubin LJ, et al. Inhaled iloprost for severe pulmonary hypertension. N Engl J Med. 2002;347(5):322–9.
5. Langleben D, Christman BW, Barst RJ, Dias VC, Galie N, Higenbottam TW, et al. Effects of the thromboxane synthetase inhibitor and receptor antagonist terbogrel in patients with primary pulmonary hypertension. Am Heart J. 2002;143(5):4A–10A.
6. Simonneau G, Barst RJ, Galie N, Naeije R, Rich S, Bourge RC, et al. Continuous subcutaneous infusion of treprostinil, a prostacyclin analogue, in patients with pulmonary arterial hypertension: a doubleblind, randomized, placebo-controlled trial. Am J Respir Crit Care Med. 2002;165(6):800–4.
7. Barst RJ, McGoon M, McLaughlin V, Tapson V, Oudiz R, Shapiro S, et al. Beraprost therapy for pulmonary arterial hypertension. J Am Coll Cardiol. 2003;41(12):2119–25.
8. Barst RJ, Langleben D, Frost A, Horn EM, Oudiz R, Shapiro S, et al. Sitaxsentan therapy for pulmonary arterial hypertension. Am J Respir Crit Care Med. 2004;169(4):441–7.
9. Galie N, Ghofrani HA, Torbicki A, Barst RJ, Rubin LJ, Badesch D, et al. Sildenafil citrate therapy for pulmonary arterial hypertension. N Engl J Med. 2005;353(20):2148–57.
10. Wilkins MR, Paul GA, Strange JW, Tunariu N, Gin-Sing W, Banya WA, et al. Sildenafil versus endothelin receptor antagonist for pulmonary hypertension (seraph) study. Am J Respir Crit Care Med. 2005;171(11):1292–7.
11. Galie N, Beghetti M, Gatzoulis MA, Granton J, Berger RM, Lauer A, et al. Bosentan therapy in patients with Eisenmenger syndrome: a multicenter, double-blind, randomized, placebo-controlled study. Circulation. 2006;114(1):48–54.
12. McLaughlin VV, Oudiz RJ, Frost A, Tapson VF, Murali S, Channick RN, et al. Randomized study of adding inhaled iloprost to existing bosentan in pulmonary arterial hypertension. Am J Respir Crit Care Med. 2006;174(11):1257–63.
13. Badesch DB, Hill NS, Burgess G, Rubin LJ, Barst RJ, Galie N, et al. Sildenafil for pulmonary arterial hypertension associated with connective tissue disease. J Rheumatol. 2007;34(12):2417–22.
14. Simonneau G, Rubin LJ, Galie N, Barst RJ, Fleming TR, Frost AE, et al. Addition of sildenafil to long-term intravenous epoprostenol therapy in patients with pulmonary arterial hypertension: a randomized trial. Ann Intern Med. 2008;149(8):521–30.
15. Galie N, Rubin L, Hoeper M, Jansa P, Al-Hiti H, Meyer G, et al. Treatment of patients with mildly symptomatic pulmonary arterial hypertension with bosentan (early study): a double-blind, randomised controlled trial. Lancet. 2008;371(9630):2093–100.
16. Galie N, Brundage BH, Ghofrani HA, Oudiz RJ, Simonneau G, Safdar Z, et al. Tadalafil therapy for pulmonary arterial hypertension. Circulation. 2009;119(22):2894–903.
17. Jing Z-C, Yu Z-X, Shen J-Y, Wu B-X, Xu K-F, Zhu X-Y, et al. Vardenafil in pulmonary arterial hypertension: a randomized, double-blind, placebocontrolled study. Am J Respir Crit Care Med. 2011;183(12):1723–9.
18. Zeng W-J, Xiong C-M, Zhao L, Shan G-L, Liu Z-H, Xue F, et al. Atorvastatin in pulmonary hypertension (APATH) study. Eur Respir J. 2012;40(1):67–74.
19. Fukumoto Y, Yamada N, Matsubara H, Mizoguchi M, Uchino K, Yao A, et al. Double-blind, placebo-controlled clinical trial with a rho-kinase inhibitor in pulmonary arterial hypertension. Circ J. 2013;77(10):2619–25.
20. Ghofrani H-A, Galie N, Grimminger F, Grunig E, Humbert M, Jing Z-C, et al. Riociguat for the treatment of pulmonary arterial hypertension. N Engl J Med. 2013;369(4):330–40.
21. Simonneau G, Torbicki A, Hoeper MM, Delcroix M, Karlocai K, Galie N, et al. Selexipag: an oral, selective prostacyclin receptor agonist for the treatment of pulmonary arterial hypertension. Eur Respir J. 2012;40(4):874–80.
